# Brusatol, a Nrf2 Inhibitor Targets STAT3 Signaling Cascade in Head and Neck Squamous Cell Carcinoma

**DOI:** 10.3390/biom9100550

**Published:** 2019-09-30

**Authors:** Jong Hyun Lee, Shobith Rangappa, Chakrabhavi Dhananjaya Mohan, Basappa, Gautam Sethi, Zhi-Xiu Lin, Kanchugarakoppal S. Rangappa, Kwang Seok Ahn

**Affiliations:** 1 College of Korean Medicine, Kyung Hee University, #47, Kyungheedae-gil, Dongdaemoon-gu, Seoul 130-701, Korea; 88mirue@gmail.com; 2 Adichunchanagiri Institute for Molecular Medicine, BG Nagara-571448, Nagamangala Taluk, Mandya District 571448, India; shobithrangappa@gmail.com; 3 Department of Studies in Molecular Biology, University of Mysore, Manasagangotri, Mysore 570006, India; cd.mohan@yahoo.com; 4 Laboratory of Chemical Biology, Department of Studies in Organic Chemistry, University of Mysore, Manasagangotri, Mysore 570006, India; salundibasappa@gmail.com; 5 Department of Pharmacology, Yong Loo Lin School of Medicine, National University of Singapore, Singapore 117600, Singapore; phcgs@nus.edu.sg; 6 Faculty of Medicine, The Chinese University of Hong Kong, Rm 101, 1/F Li Wai Chun Building, CUHK, Shatin, N.T. 999077, Hong Kong; linzx@cuhk.edu.hk; 7 Institution of Excellence, Vijnana Bhavan, University of Mysore, Manasagangotri, Mysore 570006, India

**Keywords:** brusatol, STAT3 inhibitor, apoptosis, HNSCC

## Abstract

STAT3 is a latent transcription factor that plays a vital role in the transmission of extracellular signal from receptors to the nucleus. It has been regarded as a master transcription factor due to its role in the regulation of a broad spectrum of genes, which can contribute to oncogenesis. Persistent activation of STAT3 and deregulation of its signaling has been observed in various human cancers including head and neck squamous cell carcinoma (HNSCC). In the present work, we identified brusatol (BT) as a potential blocker of STAT3 signaling pathway in diverse HNSCC cells. The data from the cell-based experiments suggested that BT-induced cytotoxicity and abrogated the activation of STAT3 and that of upstream kinases such as JAK1, JAK2, and Src. It reduced the levels of nuclear STAT3 and its DNA binding ability. BT treatment increased annexin-V-positive cells, promoted procaspase-3 and PARP cleavage, and downregulated the mRNA and protein expression of diverse proteins (Bcl-2, Bcl-xl, survivin) in HNSCC cells. Taken together, brusatol can function as a promising inhibitor targeting STAT3 signaling pathway in HNSCC.

## 1. Introduction

Head and neck cancer is a diverse set of cancers that stems predominantly from the squamous cell lining of mouth, throat, and nose and referred as head and neck squamous cell carcinoma (HNSCC) [1]. It accounts for approximately 4% of cancers and over 500,000 new cases are reported annually worldwide [2,3]. Smoking, tobacco chewing, alcohol intake, and human papillomavirus infections are identified as potential risk factors of HNSCC [4]. Surgical procedures such as transoral robotic surgery, laser surgery, and non-surgical options such as radiation therapy along with chemotherapy are the choice of treatment for managing HNSCC [5].

STAT3 is an important transcription factor that can modulate the expression of genes that can regulate cell proliferation, inflammation, angiogenesis, apoptosis, and metastasis [6,7,8]. Aberrant STAT3 activity has been reported in various human malignancies such as head and neck, liver, breast, brain, kidney, bladder, pancreas, prostate cancers, and leukemias [9,10,11,12,13]. STAT3 is phosphorylated at Tyr-705 by upstream kinases (JAKs and Src) in response to extracellular stimulation with cytokines (IL-6 family) and growth factors (EGF, PDGF) [14,15,16]. Moreover, hyperactivation of STAT3 has been linked with chronic inflammation and malignant transformation [17,18,19] and overexpression of STAT3 has been linked with the negative prognosis in human malignancies [20,21,22,23]. Collectively, these pieces of evidence suggest, STAT3 as one of the major contributing factors in the initiation and progression of cancer and blockade of STAT3 signaling can be a suitable therapeutic approach to treat human cancers.

BT is a quassinoid, initially isolated from the seeds of *Brucea sumatrana* in 1968 and has been extensively studied for its antitumor potential in several cancer models [24]. Prior investigations have identified nuclear factor erythroid 2-related factor-2 (Nrf-2), a redox sensitive transcription factor as the major cellular target of BT [25]. BT has also been reported to sensitize cancer cells to carboplatin, 5-fluorouracil, gemcitabine, etoposide, and paclitaxel by abrogating Nrf-dependent defense system [25,26]. It was also demonstrated that gefitinib-resistant NSCLC (HCC827GRKU) cells were at least seven times more sensitive to BT than its gefitinib-sensitive counterpart (HCC827) [27]. BT can augment the responsiveness of lung cancer cells to ionizing radiation by increasing the levels of reactive oxygen species and causing DNA damage [28]. In contrast, Vartanian and colleagues showed that the action of BT may not be only restricted to its effect on Nrf-2, instead it can abrogate global protein synthesis [29]. BT also induced the degradation of HIF-1α mediated by the activation of prolyl hydroxylases. They also reported that BT suppressed c-Myc expression and overexpression of c-Myc blocked brusatol-driven HIF-1α degradation [30]. In another report, BT was found to activate JNK and p38 MAPK pathways with concurrent inhibition of proinflammatory signaling pathways such as NF-κB and STAT3 in pancreatic cancer cells [31]. In the present investigation, we tested the effect of BT on the constitutive STAT3 signaling cascade in HNSCC cell lines. The findings established that BT can act as a potent inhibitor of STAT3 signaling in different HNSCC cell lines.

## 2. Materials and Methods

### 2.1. Reagents

Brusatol (BT) was provided by Professor Zhi-Xiu Lin. The stock solution of BT (10 mM) was prepared in dimethyl sulfoxide, stored at −80 ℃, and diluted in cell culture medium for use. Dimethyl sulfoxide (DMSO), 3-(4,5-Dimethylthiazol-2-yl)-2,5-diphenyltetrazolium bromide (MTT), Sodium dodecyl sulfate (SDS), and ribonuclease A from bovine pancreas were purchased from Sigma–Aldrich (St. Louis, MO, USA). Bovine serum albumin was purchased from Biosesang (Sungnam, Korea). RPMI1640, DMEM/low, MEM media, fetal bovine serum (FBS), and antibiotic-antimycotic mixture were obtained from Thermo Scientific HyClone (Waltham, MA, USA). FITC Annexin V Apoptosis Detection Kit I was purchased from BD Biosciences (San Diego, CA, USA). Caspase-3 inhibitor Z-DEVD-FMK was purchased from Calbiochem (San Diego, CA, USA).

### 2.2. Cell Lines and Culture Conditions

HNSCC cell lines UMSCC 47 (HPV-16-positive squamous carcinoma cell line), UD SCC2 (HPV16-positive hypopharyngeal carcinoma cell line), JMAR (squamous cell carcinoma from the floor of mouth), Tu167 (floor of mouth squamous cell carcinoma line), LN686 (lymph node metastasis tumor cells), and FaDu (squamous cell carcinoma from hypopharynx) were provided by Prof. Sang-Wook Lee (Ulsan College of Medicine, Asan Medical Center, Seoul, Korea). YD-10B (oral squamous carcinoma) and HN-9 (established from an undifferentiated carcinoma of the parotid gland) were purchased from Korean cell line bank (Seoul, Korea). Normal adult human primary epidermal keratinocytes HaCaT cells were obtained from the American Type Culture Collection (Manassas, VA, USA). All cells were cultured in medium containing 10% FBS and 1% P/S. Cells were maintained at 37 °C in a 5% CO_2_ atmosphere. At ~70–90% confluence, the cells were - using 0.05% trypsin/EDTA. In all the experiments, DMSO was used as a vehicle control.

### 2.3. Preparation of Whole-Cell Lysates

For the detection of expression of proteins, BT-treated whole-cell lysates were prepared as reported previously [32,33,34] using a lysis buffer [Tris (20 mM, pH 7.4), NaCl (250 mM), EDTA (2 mM, pH 8.0), Triton X-100 (0.1%), aprotinin (0.01 mg/mL), leupeptin (0.005 mg/mL), phenylmethane sulfonyl fluoride (0.4 mM), and NaVO_4_ (4 mM)].

### 2.4. Western Blot Analysis

The protein concentration was estimated in cell lysates and western blot analysis was done as reported earlier [35,36,37].

### 2.5. Electrophoretic Mobility Shift Assay (EMSA)

EMSA was performed to analyze the interaction of STAT3-DNA as described previously [38,39]. Briefly, cells were subjected to BT treatment (5 nM for 4 h) and the nuclear extract was prepared. The protein-oligonucleotide complex was subjected to PAGE and blotted to a nylon membrane followed by cross-linkage with UV.

### 2.6. Immunocytochemistry for the Distribution of STAT3

The distribution of phosphorylated STAT3 in the cells were analyzed as described earlier [40].

### 2.7. Monitoring of Cell Growth with the RTCA DP Instrument

The growth of UD SCC2, JMAR, and YD-10B cells constantly assessed for 48 h using the xCELLigence RTCA DP Instrument (Roche Diagnostics GmbH, Mannheim, Germany).

### 2.8. Annexin V Assay

Apoptosis-inducing effect of BT on HNSCC cells was evaluated using Annexin assay as described previously [35].

### 2.9. Measurement of Intracellular Reactive Oxygen Species

YD-10B cells were treated with 5 nM of BT 48 h. Then, the cells were washed twice, stained with 10 μM of 2,7-dichlorofluorescin-diacetate (DCFH-DA) for 20 min and washed twice. The esterified form of DCFH-DA can permeate cell membranes and thereafter deacetylated by intracellular esterases. The resulting compound, dichlorofluorescin (DCFH), reacts with hydrogen peroxide or ROS to form the fluorescent compound, dichlorofluorescin (DCF) [41]. The amount of intracellular fluorescent DCF was measured by BD Accuri C6 plus flow cytometer (BD Biosciences, San Diego, CA, USA). Acquisition and analysis of the data were performed using BD Accuri C6 plus software (version 1.0.23.1).

### 2.10. In Silico Analysis

Accelrys software called Discovery Studio was used for the molecular docking purposes. Crystal structure of STAT-3β homodimer (PDB ID: 1BG1) was used to understand the structure-based interaction with BT. LIGANDFIT protocol was used for the docking analysis. Initially, the three-dimensional protein template was cleaned, and the spatial orientation of the active site was identified in the SH2 domain of STAT3. Using the CHARMM force field, the protein energy minimizations were performed. The ligand such as BT was docked and the final docking position was evaluated using the interaction scoring function in the LIGANDFIT module of Discovery Studio. The Discovery Studio 2.5 visualization tool is used to analyze the 10 top hit conformations.

### 2.11. Statistical Analysis

Data was expressed as the mean ± S.D. In all figures, vertical error bars denote S.D. The significance of differences between groups was evaluated by Student’s *t*-test and one-way analysis of variance, (ANOVA) test. A *p*-value of less than 0.05 was considered statistically significant.

## 3. Results

### 3.1. BT Reduced the Cell Viability of HNSCC Cells

**Figure 1:**
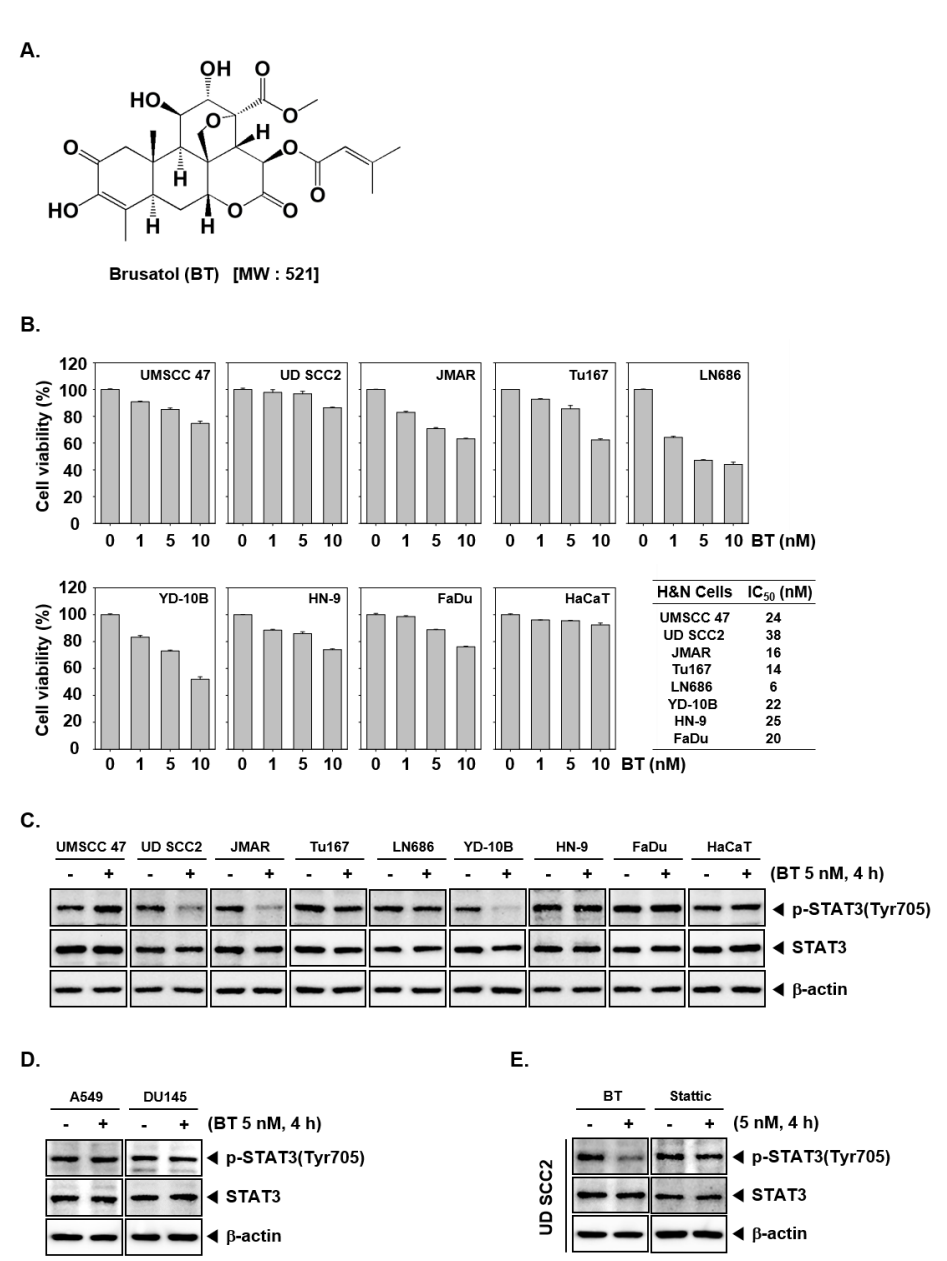
Brusatol (BT) suppresses cell viability and selectively blocked the activation of STAT3 in head and neck squamous cell carcinoma (HNSCC). (**A**) The chemical structure of Brusatol (BT). (**B**) HNSCC cells (UMSCC 47, UD SCC2, JMAR, Tu167, LN686, YD-10B, HN-9, and FaDu) and HaCaT cells were treated with various concentration of BT for 24 h, and cell viability was determined by 3-(4,5-Dimethylthiazol-2-yl)-2,5-diphenyltetrazolium bromide (MTT) assay. (**C**) HNSCC cells (UMSCC 47, UD SCC2, JMAR, Tu167, LN686, YD-10B, HN-9, and FaDu) and HaCaT cells were treated with 5 nM of BT for 4 h. Thereafter, equal amounts of lysates were analyzed by western blot analysis. β-actin was used as an input control to verify equal protein loading. (**D**) A549 and DU145 cells were treated with 5 nM of BT for 4 h. Thereafter, equal amounts of lysates were examined by western blot analysis. (**E**) UD SCC2 cells were treated with 5 nM of BT or static for 4 h. Thereafter, equal amounts of lysates were examined by western blot analysis.

The chemical structure of BT is presented as Figure 1A. We initially explored the effect of BT on the viability in a panel of HNSCC cells such as UMSCC 47, UD SCC2, JMAR, Tu167, LN686, YD-10B, HN-9, and FaDu using MTT assay. These cells were treated with different doses (1, 5, or 10 nM) of BT for 24 h and cell viability was measured. We observed the reduction in cell viability on treatment with BT in a dose-dependent manner in all the tested cells (Figure 1B). We also deciphered the effect of BT on normal HaCaT cells and did not observe any substantial cytotoxicity at any tested dosage. The IC_50_ values for each cell line can be found in the box on the right (Figure 1B).

### 3.2. BT Inhibited Constitutive STAT3 Phosphorylation in UD SCC2, JMAR, YD-10B Cells

Constitutive phosphorylation of STAT3 at Tyr705 has been encountered in several types of cancer cells and is responsible for the dimerization of phosphorylated STAT3 and its subsequent nuclear translocation. Further, we examined the result of BT exposure on the constitutive phospho-STAT3 levels in UMSCC 47, UD SCC2, JMAR, Tu167, LN686, YD-10B, HN-9, FaDu, and HaCaT cells. Figure 1C demonstrated that all the tested HNSCC cell lines show constitutive STAT3 phosphorylation. Interestingly, BT inhibited STAT3 (Tyr705) activation only in UD SCC2, JMAR, YD-10B cells, and the levels of total STAT3 protein content were not affected (Figure 1C). These results suggest that, BT may interfere with the STAT3 dimerization and subsequent events by downregulation of constitutive phosphorylation.

### 3.3. BT Failed to Suppress STAT3 Activation in A549 and DU145 Cells

We next investigated whether BT could modulate constitutive STAT3 activation in human prostate and lung cancer cell lines. Because A549 and DU145 cells can express constitutive STAT3 activation, we investigated whether BT could also target STAT3 phosphorylation in these cells. Interestingly it was noted that BT did not inhibit STAT3 activation in A549 and DU145 cells (Figure 1D).

### 3.4. BT Mitigated STAT3 Activation More Effectively than Stattic in UD SCC2 Cells

We next compared the STAT3 inhibitory efficacy of BT with stattic, a selective small molecule STAT3 inhibitor at the same concentration. The results demonstrated that stattic inhibited STAT3 at 5 nM in UD SCC2 cells, but the effect was less pronounced when compared to BT (Figure 1E), thereby suggesting that BT may act as a more potent inhibitor of STAT3 signaling than stattic.

### 3.5. BT Abrogated DNA Binding Ability of STAT3

**Figure 2:**
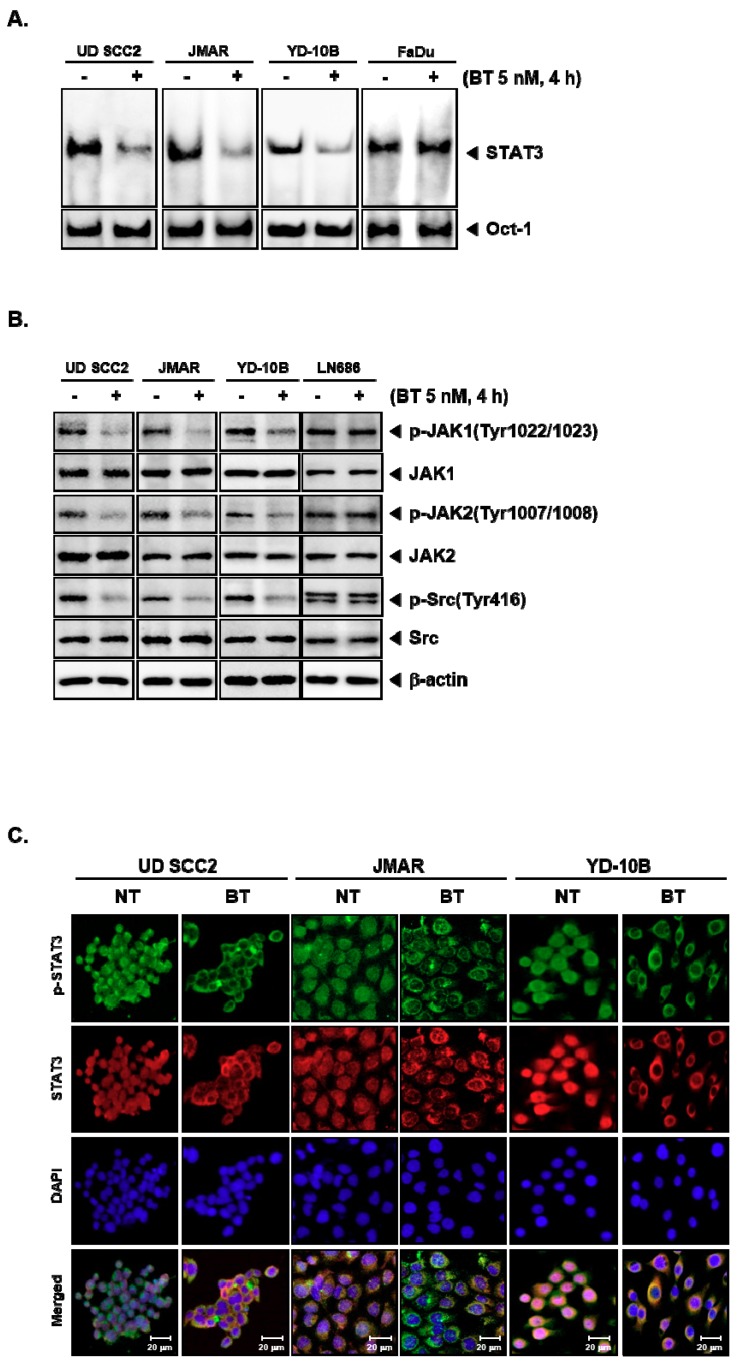
BT downregulates upstream signaling pathway of STAT3. (**A**) Nuclear extracts from non-treated (NT) HNSCC (UD SCC2, JMAR, YD-10B, and FaDu) or treated with 5 nM of BT for 4 h were tested for DNA binding to STAT3 by electrophoretic mobility shift assay (EMSA). Oct-1 was used as a loading control. (**B**) HNSCC cells (UD SCC2, JMAR, YD-10B, and LN686) were treated with 5 nM of BT for 4 h. And western blotting was done as indicated above. β-actin was used as an input control to verify equal protein loading. (**C**) HNSCC (UD SCC2, JMAR, YD-10B) cells were either non-treated (NT) as control or treated with 5 nM of BT. And analyzed for the intracellular distribution of p-STAT3 and STAT3 by immunocytochemistry. The nuclei are counterstained with DAPI (5 µg/mL) for 3 min.

Phosphorylation of Tyr705 is a critical event in STAT3 dimerization, nuclear localization and binding to specific response elements to induce gene transcription [42,43]. Therefore, we next determined the consequence of BT treatment on the DNA binding ability of STAT3. The EMSA results showed a significant reduction in STAT3-DNA–binding activities on treatment with BT in the nuclear extracts of UD SCC2, JMAR, YD-10B cells but not in FaDu cells (Figure 2A). These results infer that BT selectively reduces the DNA binding ability of STAT3. Oct-1 was used as a loading control.

### 3.6. BT Repressed the Constitutive Phosphorylation of Upstream Kinases

JAKs and Src are the upstream kinases that are responsible for the activation of STAT3 protein in the cytoplasm [44,45]. We probed the action of BT on the constitutive activation of JAK1, JAK2, and Src in UD SCC2, JMAR, YD-10B, and LN686 cells. We observed the significant reduction in phosphorylation of JAK1 (Tyr1022/1023), JAK2 (Tyr1007/1008), and Src (Tyr416) on treatment with BT in UD SCC2, JMAR, YD-10B cells but not in LN686 cells (Figure 2B). The total protein levels of JAK1/2 and Src were not affected. These results indicated that BT induces abrogation of STAT3 signaling by interfering with the activities of all the tested non-receptor tyrosine kinases in UD SCC2, JMAR, YD-10B cells.

### 3.7. BT Reduced the Nuclear STAT3 Level in HNSCC Cells

The phosphorylated STAT3 monomers can undergo dimerization and move into nucleus to drive the expression of target genes. We next examined the distribution of STAT3 in BT treated and untreated cells using immunocytochemistry (Figure 2C). We observed the reduction in the nuclear localization of STAT3 in UD SCC2, JMAR, YD-10B cells on BT treatment. Since there was a significant reduction in the phosphorylation of STAT3, a decline in the nuclear pool of STAT3 can also be expected. These findings are consistent with the results of the previous experiments, thus establishing the inhibitory effect of BT on STAT3 signaling cascade.

### 3.8. BT Downregulated the Protein Expression of STAT3-Driven Genes

**Figure 3:**
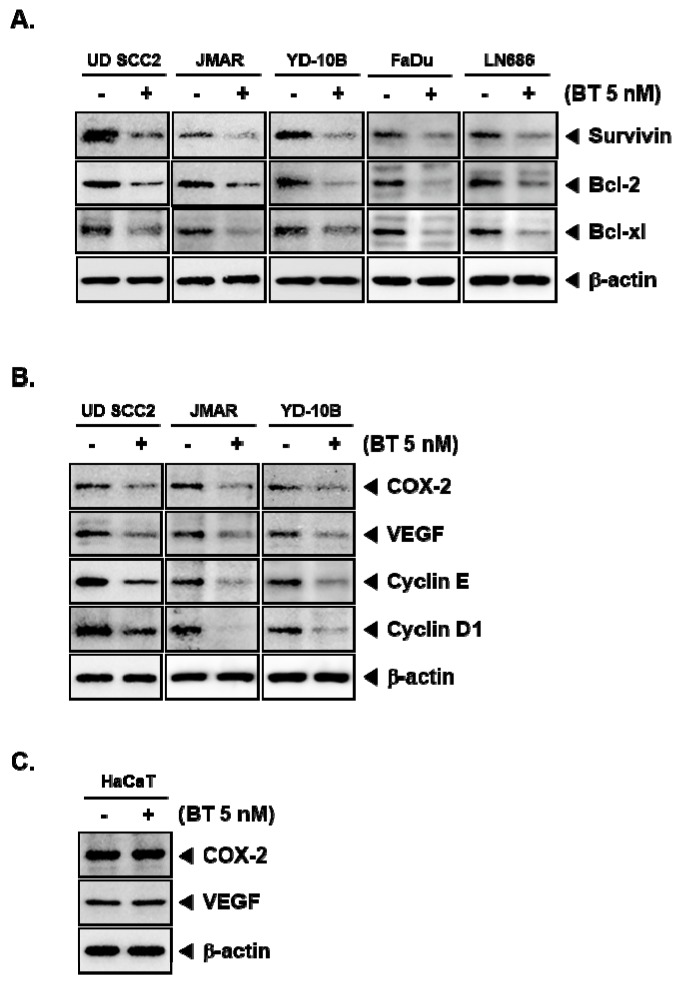
BT suppresses the STAT3-regulated target genes. (**A**) HNSCC cells (UD SCC2, JMAR, YD-10B, FaDu, and LN686) were treated with 5 nM of BT for 48 h and western blotting was performed Survivin, Bcl-2, Bcl-xl; (**B**) COX-2, VEGF, Cyclin E, and Cyclin D1 and western blotting was done. β-actin was used as an input control to verify equal protein loading. (**C**) HaCaT cells were treated with 5 nM of BT for 48 h and western blotting was performed COX-2 and VEGF. β-actin was used as an input control to verify equal protein loading.

Genes that code for the proteins which are entangled with the regulation of apoptosis, cell cycle, proliferation, inflammation including Bcl-2, Bcl-xl, COX-2, VEGF, and cyclin D1 are under transcriptional control of STAT3 [46]. Therefore, we next evaluated the effect of BT on the expression of aforementioned gene products. We found that BT substantially downregulated the protein expression of Survivin, Bcl-2, Bcl-xl, COX-2, VEGF, cyclin E, and cyclin D1 in UD SCC2, JMAR, YD-10B, FaDu and LN686 cells (Figure 3A,B). However, there was a marginal effect on the expression of gene products in HaCaT cells (Figure 3C).

### 3.9. BT Abrogated the Growth of HNSCC Cells

**Figure 4:**
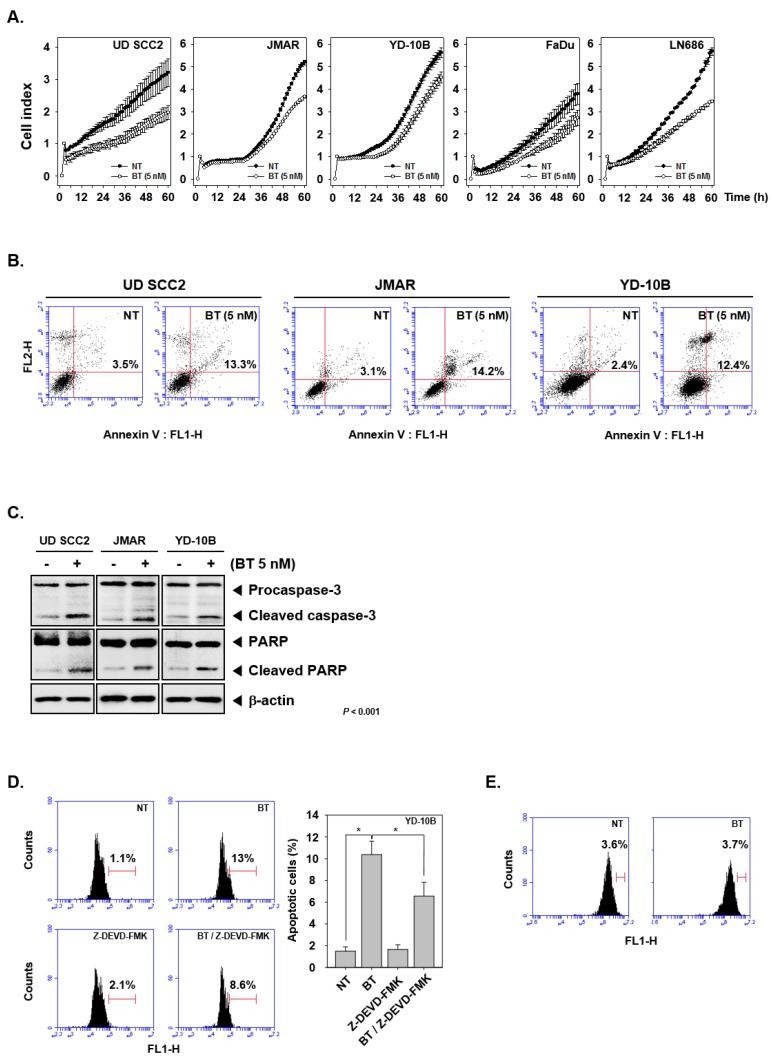
BT inhibits cell proliferation and induces apoptosis in HNSCC. (**A**) HNSCC (UD SCC2, JMAR, YD-10B, FaDu, and LN686) cells were either non-treated (NT) as control or treated with 5 nM of BT. Then continuously monitored using the Roche xCELLigence Real-Time Cell Analyzer (RTCA) DP instrument (Roche Diagnostics GmbH, Germany). (**B**) HNSCC (UD SCC2, JMAR, YD-10B) cells were treated with 5 nM of BT for 48 h. The cells were incubated with a FITC conjugated Annexin V, then examined for an early apoptotic effect with flow cytometry. (**C**) HNSCC cells (UD SCC2, JMAR, and YD-10B) were treated with 5 nM of BT for 48 h and western blotting was performed using antibodies against Caspase-3 and PARP. β-actin was used as an input control to verify equal protein loading. (**D**) YD-10B Cells were pre-treated with or without a Z-DEVD-FMK (caspase-3 inhibitor; 20 μM) 1 h before being treated with 5 nM BT for an additional 48 h. The cells were incubated with an FITC-conjugated Annexin V antibody and then analyzed by flow cytometry. The graph indicated quantitative analysis of apoptotic cells. (**E**) YD-10B cells were treated with 5 nM of BT for 48 h. Cells were incubated with 10 μM of DCFH-DA for 20 min at 37 °C then DCF fluorescence was measured by flow cytometry. * indicates p value less than 0.05.

To determine antiproliferative actions of BT on UD SCC2, JMAR, YD-10B, FaDu, and LN686 cells, we treated cells with BT (5 nM) and analyzed their viability in the intervals of every 15 min using RTCA DP Instrument as described in methods. We observed a significant mitigation in the proliferation of tested cells upon BT treatment in a time-dependent manner (Figure 4A).

### 3.10. BT Promoted Apoptotic Cell Death

The phosphatidylserine translocation to the outer leaflet of the biological membrane is the critical event observed in the cells undergoing apoptosis which can be detected using annexin V staining [47,48]. We treated UD SCC2, JMAR, YD-10B cells with 5 nM of BT for 24 h and analyzed for presence of annexin-V-positive cells and observed an augmentation of annexin-V-positive cells from 3.5% to 13.3%, 3.1% to 14.2%, and 2.4% to 12.4% compared with non-treated (NT), respectively (Figure 4B).

### 3.11. BT Regulated Caspase-Mediated Apoptosis in HNSCC Cells

Procaspase-3 is the zymogen form which undergoes proteolytic cleavage in response to proapoptotic signals to attain its active form [49,50,51]. Further, it acts on various cellular proteins including caspase-activated DNase, lamins, focal adhesion kinase and responsible for cleavage of poly (ADP-ribose) polymerase (PARP) [52,53]. We evaluated the role of BT in the activation of caspase and cleavage of PARP in treated UD SCC2, JMAR, YD-10B cells. The formation of cleaved caspase-3 is an indicator of activation of procaspase in order to get the proteolytic activity, subsequently leading to the cleavage of PARP. The results demonstrated an increase in cleavage of caspase-3 and PARP on treatment with BT (5 nM) (Figure 4C).

### 3.12. Specific Blockade of Caspase-3 Cleavage Abrogated BT-Induced Apoptosis

We next examined whether BT-induced apoptosis could be modulated by inhibition of caspase-3 using Z-DEVD-FMK (irreversible caspase-3 inhibitor). We found that the BT-induced apoptosis was suppressed by the Z-DEVD-FMK treatment, thus indicating the significance of caspase-3 activation in regulating apoptosis upon BT treatment (Figure 4D).

### 3.13. BT Induced STAT3 Inhibition May be Independent of Reactive Oxygen Species (ROS)

Several natural products such as nimbolide may abrogate STAT3 activation by increasing the production of ROS [20]. We further explored the potential role of ROS in BT mediated STAT3 inhibition in YD-10B cells. The results showed that BT treatment did not significantly alter the level of ROS in the cells as evident by flow cytometric analysis (Figure 4E).

### 3.14. In Silico Interaction Studies between BT and SH2 Domain of STAT3

**Figure 5:**
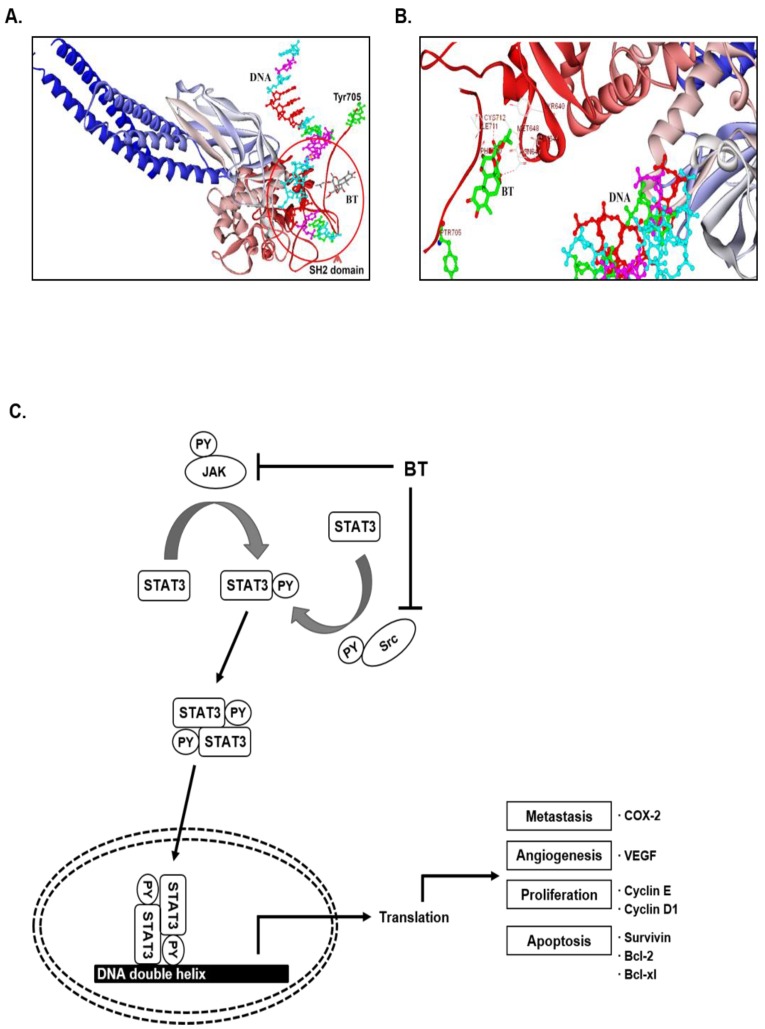
In silico interaction analysis between the SH2 domain of STAT3 and BT. (**A**) Surface view of STAT3β homodimer (PDB ID: 1BG1). (**B**) An interaction map of the SH2 domain of STAT3 with BT. The hydroxyl group of BT is involved in the formation of a hydrogen bond with Asn647 of SH2 domain and the diester side chain of BT was visualized to be sandwiched between Tyr640 and Phe710. (**C**) Schematic diagram showing the effects of BT on STAT3 signaling pathways and apoptosis.

Given the relevance with the structural similarity of previously reported JAK-STAT3 signaling inhibitors, we analyzed the probable binding mode of BT with the SH2 domain of STAT3 protein (Figure 5A). The Dock Score (DS) value for the binding of BT towards the SH2 domain of STAT3 was found to be 28.47 kcal/mole, which signifies better affinity of the molecule towards the target. We compared the binding pattern of BT with stattic, a selective inhibitor of STAT3, whose DS was found to be 19 kcal/mole, and it exhibited similar interactions with the key amino acids of SH2 domain of STAT3. The docking results presented that the hydroxyl group of BT is involved in the formation of a hydrogen bond with Asn647 of SH2 domain. The diester sidechain of BT was visualized to be sandwiched between Tyr640 and Phe710 (Figure 5B). In summary, the presence of several hydroxyl groups in BT and structural flexibility favor its interaction towards the SH2 domain of STAT3.

## 4. Discussion

STAT3 has been recognized as an attractive therapeutic target in oncology for the development of inhibitors and several agents have been identified to interfere with STAT3 signaling in various cancer models [54,55,56,57,58,59,60]. In an unstimulated cell, STAT3 remains in the cytoplasm in the unphosphorylated form and upon activation, STAT3 monomer can be phosphorylated at Tyr705 followed by its dimerization with another phosphorylated monomer to translocate into the nucleus. Persistent activation of STAT3 has been observed in a broad range of malignancies including HNSCC [61] and suppression of STAT3 activity could be a strategy to induce apoptosis and to counteract the proliferation of STAT3 positive cells [62,63,64,65]. Here, we elucidated the role of BT in regulating STAT3 signaling cascade in HNSCC cells.

BT has been reported to exhibit prominent anticancer activity in several preclinical cancer models. BT can induce its anticancer effects via modulating multiple cellular signaling events such as Nrf-2, HIF-1α, c-Myc, JNK/p38 MAPK/NF-κB/Stat3/Bcl-2 signaling pathways [31]. Xiang et al. showed that BT can downregulate the phosphorylation of STAT3 in pancreatic cancer cells but the detailed mechanism of inhibition was not deciphered [31]. The cousin compound of BT called bruceantin has also gained significant attention due to its potent antitumor activity in mouse models. However, bruceantin failed to regress tumor in phase-I/II trials and was not pursued further for clinical development [66]. Based on the reported effect of BT in the modulation of various oncogenic and proinflammatory signaling cascades, its effect on STAT3 signaling was examined in HNSCC cells.

Initially, we investigated the effect of brusatol on seven cancer cell lines (LN686, Tu167, FaDu, UMSCC47, HN-9, UD SCC2, JMAR, YD-10B) and observed a decline in the cell viability in all the tested cell lines. LN686, Tu167, JMAR, and FaDu cells displayed relatively high response rate with IC_50_ values less than 20 nM, while the others ranged between 21 to 38 nM with a relatively moderate response to BT treatment. Although, BT reduced the viability of all the tested cell lines, it failed to suppress STAT3 phosphorylation in UMSCC 47, Tu167, LN686, HN-9, and FaDu cells. As discussed in the introduction of this report, BT has been reported to induce anticancer activity via modulating diverse signaling cascades such as Nrf2, STAT3, HIF-1, c-Myc, NF-κB, JNK, and p38 pathways. Therefore, STAT3 independent cytotoxic effect of BT in UMSCC 47, Tu167, LN686, HN-9, and FaDu cells could be due to abrogation of these signaling cascades.

Several strategies can be used to terminate STAT3 signaling which encompasses the inhibition of STAT3 phosphorylation by targeting upstream kinases; blockage of STAT3 dimerization by targeting SH2 domains; interfering with nuclear export of STAT3 by targeting karyopherins; and inhibition of STAT3 mediated transcription by blocking its DNA binding domain [67]. Interestingly, our results suggested that this quassinoid BT inhibited the phosphorylation of JAK1 (Tyr1022/1023), JAK2 (Tyr1007/1008), Src (Tyr416) and STAT3 (Tyr705) indicating BT could inhibit STAT3 via modulating several upstream kinases. The pronounced inhibition of signaling cascade was documented using western blotting and EMSA studies. Immunocytochemistry results demonstrated that the reduction in nuclear STAT3 and its accumulation in the cytoplasm upon treatment with BT. This effect may be due to its interaction with the upstream kinases or direct interaction with the SH2 domain of STAT3. Although natural products such as guggulsterone, honokiol, curcumin, resveratrol, flavopiridol and cucurbitacin have been reported to inhibit STAT3 signaling in human malignancies, the precise underlying mechanism in imparting their STAT3 inhibitory activity has not been fully understood [67]. In silico analysis predicted the favorable interaction between BT and SH2 domain of STAT3, and this needs to be validated indirect binding kinetics in future studies.

Previous studies also suggest that the inhibition of STAT3 signaling can regulate apoptosis in tumor cells [68]. As a consequence of BT induced STAT3 inhibition, we observed a decline in the cell viability and increase in apoptosis at sub-micromolar concentrations. Activation of caspase-3 and cleavage of PARP are the major events observed during programmed cell death [69]. Caspase-3 is a death protease that is present in the inactive form and the relaying of the apoptotic signals from initiator caspases (caspase-8 and -9) can lead to the activation of caspase-3 by proteolytic processing [70]. The activated caspase-3 can than cleave PARP, a DNA repair enzyme into larger (89 kDa) and smaller (24 kDa) fragments [71]. The larger fragment possesses catalytic site with reduced DNA binding ability and smaller fragment has a DNA binding domain [72]. The larger fragment moves to cytoplasm upon cleavage; whereas the smaller fragment irreversibly binds to nicked DNA and prevents repair of DNA which can drive the cell towards apoptosis [72]. We observed both the cleavage of caspase-3 and PARP upon treatment with BT in HNSCC cells.

STAT3 can modulate the expression of numerous genes with diverse biological effects including Bcl-2 family proteins, survivin, COX-2, VEGF, and cyclin D1 [46,73]. Bcl-2 and Bcl-xl are apoptosis suppressor proteins that can interact with pro-apoptotic proteins in order to modulate the process of programmed cell death and transform cells [74]. COX-2 can regulate cancer cell survival and proliferation, inflammation, metastasis, angiogenesis, and resistance to chemotherapy [75,76,77]. Survivin belongs to the inhibitor of apoptosis (IAP) protein family and imparts its activity by blocking caspases. High survivin expression has been observed in cancers and its expression may be associated with poor clinical outcome [78]. VEGF is a major growth factor that controls the angiogenesis by interacting with VEGFR [79] and the VEGFR signaling has been identified as negative prognostic factor which can be associated with pathological angiogenesis in human malignancies [79,80]. Cyclin D1 and E are the cell cycle regulators that are crucial for G1 to S phase transition with their respective cyclin-dependent kinase binding partners. Overexpression of cyclin D1, and cyclin E has been identified to provide proliferative advantage to the tumor cells [81,82]. Interestingly, BT downregulated the protein expression of Bcl-2, Bcl-xl, survivin, COX-2, VEGF, cyclin D1, and cyclin E.

Overall, aberrant activity STAT3 have been described as a contributing factor for the formation of a tumor, chemoresistance, and poor prognosis in HNSCC [83]. In addition to Nrf-2 modulatory activity, we have explored another underlying molecular mechanism of BT to impart its antitumor activity in tumor cells that display constitutively active STAT3. In conclusion, these findings comprehensively demonstrate that BT may act as an effective inhibitor of STAT3 signaling and it can be a promising drug to test in other STAT3 overexpressing preclinical cancer models.
